# Palliative extubation in pediatrics: a scoping review

**DOI:** 10.1016/j.jped.2025.101468

**Published:** 2025-11-12

**Authors:** Carolina de Araújo Affonseca, Luís Fernando Andrade de Carvalho, Lêni Márcia Anchieta

**Affiliations:** a Unimed Belo Horizonte, Belo Horizonte, MG, Brazil; b Unidade de Terapia Intensiva Pediátrica da Santa Casa de Misericórdia de Belo Horizonte, Belo Horizonte, MG, Brazil; c Unidade de Terapia Intensiva Pediátrica do Hospital Mater Dei Contorno, Belo Horizonte, MG, Brazil; d Departamento de Pediatria da Universidade Federal de Minas Gerais, Belo Horizonte, MG, Brazil

**Keywords:** Terminal care, Respiration, Artificial, Withholding treatment, Airway extubation, Palliative care, Pediatrics

## Abstract

**Objective:**

To evaluate evidence in the literature on palliative extubation in pediatrics in the context of palliative care, in any healthcare setting, to synthesize knowledge, identify gaps, and highlight future research opportunities.

**Data sources:**

The PRISMA-ScR recommendations and the JBI Collaboration method were used. Searches were conducted in: Virtual Health Library, PubMed, Scopus, Embase, Cochrane Library and Web of Science. The following strategy was used: Population - children and adolescents (0 to 18 years) undergoing invasive mechanical ventilation; Concept - practices, experiences, or approaches related to palliative extubation; Context - palliative care in a hospital, hospice or home setting. Original articles published up to April 2025 were included; those that didn’t define age or were over 18 years, opinion pieces, editorials, and conference proceedings were excluded. Two independent reviewers extracted the data; discrepancies were resolved by consensus or with a third reviewer. The quality of the studies was assessed using the critical appraisal tools recommended by the JBI.

**Data synthesis:**

Twelve articles were selected: eight case reports and four cross-sectional studies, totaling 129 patients; 128 were analyzed. In 78.1% of cases, palliative extubation was performed in a hospital setting, mainly in the ICU (72.6%); 93% used an endotracheal tube; 95.3% received analgesia/sedation around the time of extubation; 90.6% died after support withdrawn.

**Conclusions:**

Knowledge of practices, experiences, and challenges related to palliative extubation in pediatrics is essential to support clinical decision-making and ensure that it is performed in a timely, responsible, and technically appropriate manner, following the principles of palliative care.

## Introduction

Progressive advances in healthcare in recent decades, while promoting clinical recovery and increased survival of children and adolescents affected by serious and life-threatening diseases, have brought about significant changes in the epidemiological profile of health conditions, leading to an increase in the number of patients with chronic conditions, often dependent on artificial life support [1, 2, 3, 4, 5, 6, 7]. It is estimated that one in four children is affected by at least one chronic disease, often with high morbidity and mortality, including reduced self-reported quality of life, neurocognitive impairment, and functional impairment [6, 7, 8, 9].

It is usually assumed that offering advanced life-sustaining resources is the most appropriate course of action in situations that threaten life. However, there are occasions when not initiating or discontinuing life-sustaining measures is ethically appropriate and even recommended, as the suffering associated with treatment outweighs its potential benefits [10,11]. The planned withdrawal of life support measures can be defined as the process in which medical interventions such as the use of vasopressors or invasive mechanical ventilation (IMV), for example, are discontinued in order to allow the patient to die at their own time, naturally, and as a result of the disease that afflicts them and/or its complications. Withdrawal of life support in this context reflects the transition from an invasive and interventionist clinical approach to one with greater emphasis on patient comfort and family care [12,13].

The decision to discontinue life-sustaining therapies in pediatrics involves complex ethical, clinical, and family considerations; it should be based on therapeutic proportionality and an assessment of expected quality of life. Withdrawal of advanced life support is a delicate and individualized process, centered on the child and family. The most used procedures vary according to the type of support involved and the goals of care.

Thus, it is important to further discuss palliative extubation, a procedure that consists in discontinuing the use of IMV of patients with severe and irreversible diseases, when the defined goal of care is to provide care and confort in a shared decision between the healthcare team, the family, and, whenever possible, the patient, allowing the disease to follow it’s natural course until death [1,10].

Studies describe the step-by-step procedure for palliative extubation, associated symptoms, main treatments instituted, and most frequent outcomes in adult patients [1,14, 15, 16, 17, 18]. There are few studies in pediatrics, [19] and a literature review may improve understanding of aspects related to palliative extubation in this age group, providing scientific support and safety to health professionals working in pediatric intensive care and palliative medicine. Currently, there is a scoping review in the literature that refers to recommendations from a technical and humanistic perspective for the implementation of palliative extubation in pediatrics; [19] evaluates parental experience related to the death of children in the pediatric intensive care unit (PICU); [20] and addresses the process of death in children after the planned withdrawal of life support measures in the PICU [21].

This article describes a scoping review related to palliative extubation in pediatrics in any healthcare setting to summarize the available knowledge, identify possibilities for improvement, and highlight future research opportunities.

## Methods

Preferred Reporting Items for Systematic Reviews and Meta-Analysis for Scoping Reviews (PRISMA-ScR) [22] and the method proposed by the JBI Collaboration [23] were used for this review, registered in the Open Science Framework (https://doi.org/10.17605/OSF.IO/7C6B5). The research question was “What scientific evidence is available on pediatric patients with serious and irreversible illnesses undergoing palliative extubation in any care setting?” The “Population, Concept, Context (PCC)” strategy was used, in which: P - children and adolescents (0 to 18 years) undergoing IMV; C - practices, experiences, or approaches related to palliative extubation; C - palliative extubation performed in a hospital, hospice, or home setting.

The search for studies was conducted by consulting the Virtual Health Library (VHL), PubMed (Medline), Scopus, Embase, Cochrane Library, and Web of Science databases. The following keywords were selected: artificial respiration; terminal care; terminally ill patient; medical futility; treatment withdrawal; life-prolonging care; extubation; palliative care. Other terms commonly used in the literature were included in the search strategies: palliative extubation; compassionate extubation; terminal withdrawal; terminal extubation; terminal weaning. The search strategies are presented in Table 1.

**Table 1:** Search strategies in the selected databases for the Scoping Review (up to April 2025).

DATABASES	STRATEGIES
Biblioteca Virtual em saúde	("Cuidados Paliativos" OR "Palliative Care" OR "Soins palliatifs") AND (Extubação OR "Airway Extubation" OR "Extubación Traqueal" OR Extubation OR "Respiração Artificial" OR "Respiration, Artificial" OR "Respiración Artificial" OR "Ventilation artificielle" OR "Extubação Paliativa" OR "Extubação Compassiva" OR "Extubação Terminal" OR "Palliative Extubation" OR "Compassionate Extubation" OR "Terminal Withdrawal" OR "Terminal Extubation" OR "Terminal Weaning" OR "Artificial Respiration")
Medline	("Palliative Care") AND ("Airway Extubation" OR "Respiration, Artificial" OR "Palliative Extubation" OR "Compassionate Extubation" OR "Terminal Withdrawal" OR "Terminal Extubation" OR "Terminal Weaning" OR "Artificial Respiration")
Cochrane	("Palliative Care") AND ("Airway Extubation" OR "Respiration, Artificial" OR "Palliative Extubation" OR "Compassionate Extubation" OR "Terminal Withdrawal" OR "Terminal Extubation" OR "Terminal Weaning" OR "Artificial Respiration")
Scopus	("Palliative Care") AND ("Airway Extubation" OR "Respiration, Artificial" OR "Palliative Extubation" OR "Compassionate Extubation" OR "Terminal Withdrawal" OR "Terminal Extubation" OR "Terminal Weaning" OR "Artificial Respiration")
Web of Science	("Palliative Care") AND ("Airway Extubation" OR "Respiration, Artificial" OR "Palliative Extubation" OR "Compassionate Extubation" OR "Terminal Withdrawal" OR "Terminal Extubation" OR "Terminal Weaning" OR "Artificial Respiration")
Embase	('palliative therapy') and (extubation)

Two independent researchers selected studies that met the following criteria: published up to April 2025 and in any language, provided that the abstract was available in English. The title and abstract were initially evaluated to confirm that they addressed the research question and met the previously established inclusion criteria. When necessary, the study was read in its entirety. Those that did not define age or describe patients over 18 years of age, unpublished studies, or publications in conference proceedings, editorials, and opinion articles were excluded.

For data extraction, two independent researchers conducted a comprehensive review of the preselected studies. When there was disagreement between the two researchers regarding the inclusion of an article, a third author read the article independently and decided whether or not to include it in the review.

Data extraction included author details, country of origin, study design, objectives, sample characteristics, methodology, and results. The results were organized according to their contribution to evaluating the outcomes proposed in the review; the variables extracted were: location where the palliative extubation procedure was performed; device used to provide IMV (endotracheal tube or tracheostomy cannula); time of IMV use until palliative extubation; time elapsed between decision-making and palliative extubation; medications used in preparation for palliative extubation; signs/symptoms after palliative extubation and treatment instituted for their control; patient outcomes: discharge or death.

The Joanna Briggs Institute (JBI) critical appraisal tools [23] were used to characterize the quality of the studies and risk of bias according to the type of study. Based on the recommendations of this tool, the authors evaluated the articles and classified them as high, moderate, or low quality.

## Results

The main results were categorized according to variables of interest associated with PCC to organize them systematically. For each variable, the results were subdivided into three types of analysis: “All Studies”, “Case Reports”, and “Cross-sectional Studies”, since the study design may influence the results.

### Included studies and their characteristics

**Figure 1:**
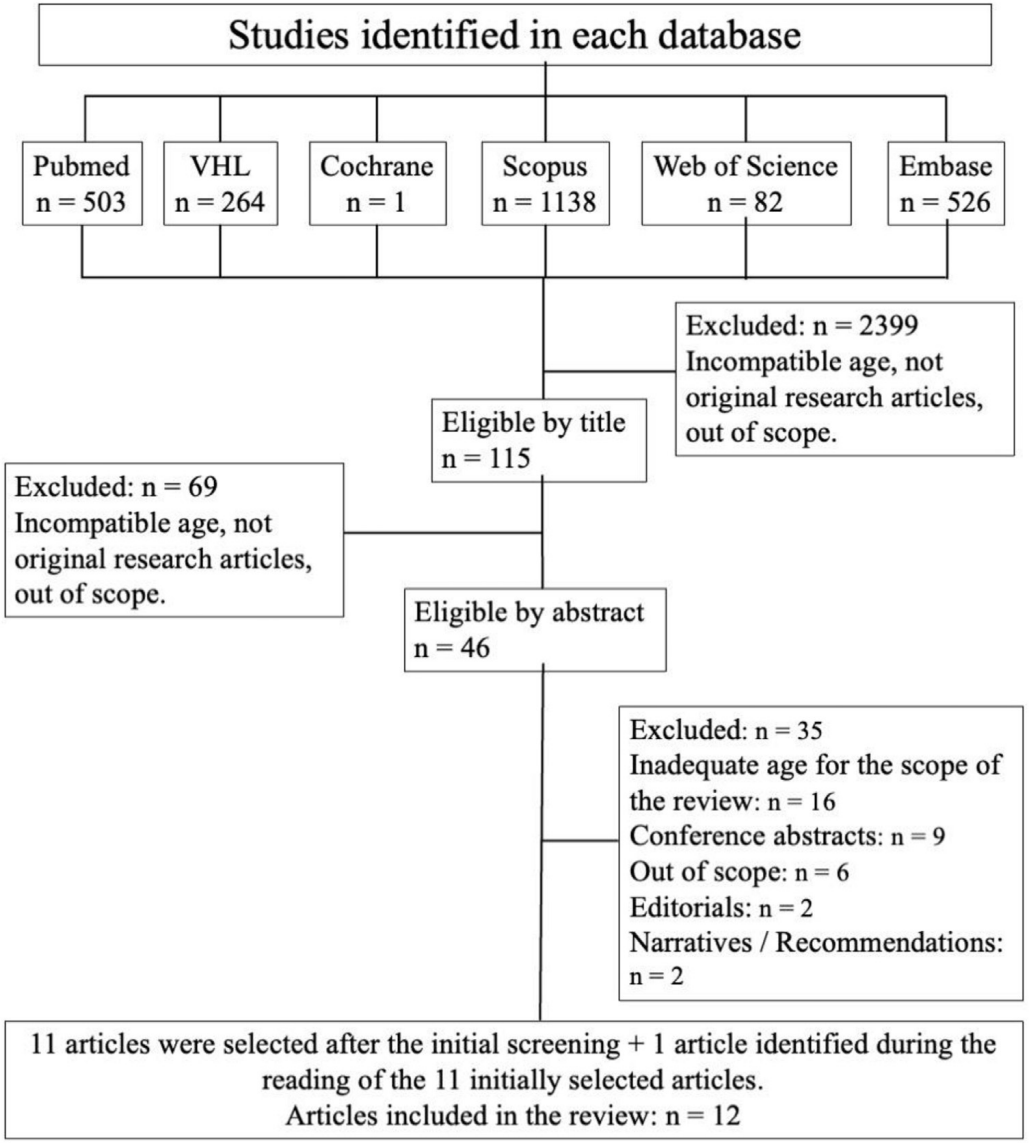
Scoping Review Flowchart – Pediatric Palliative Extubation (up to April 2025).

The initial search of the databases resulted in 2,514 documents: 115 were preselected, and 46 were eligible for full-text reading. According to the PCC question, 11 articles were included in this review [24, 25, 26, 27, 28, 29, 30, 31, 32, 33, 34]. After reading the bibliographic references of these articles, one more article was added, [35] totaling 12 articles, as shown in Figure 1.

From the 12 selected studies, variables of interest related to palliative extubation in pediatrics, in the age group from zero to 18 years, in any care setting, were extracted and analyzed (Table 2).

**Table 2:** Data extracted from the 12 eligible articles published between 1994 and 2025.

Title	Author and year of publication	Country of study	Type of study	Objectives	Population	Results
Withdrawal of neonatal intensive care in the home	Hawdon JM et al. (1994)[35]	United Kingdom	Case report	To present the report of patients undergoing palliative extubation at home.	Three patients with malformations and/or complications of prematurity transferred from the neonatal ICU to undergo palliative extubation at home.	**Patient 1**: female, 19 weeks old (7 weeks corrected age), 28 weeks premature. BIR and PN: 570 g. Dysmorphisms secondary to an undiagnosed syndrome, right diaphragmatic hernia. After 10 unsuccessful attempts at extubation, transferred for extubation at home. After extubation, she received O_2_ via a face mask. Death occurred within 8 hours. **Patient 2**: female, 30 weeks premature due to placental abruption. Acute fetal distress. PN: 1500 g. Mechanical ventilation since birth. Bilateral intraventricular and intraparenchymal hemorrhage and hydrocephalus. At 7 weeks of life, worsening ventilation and signs of portal vein hypertension with hepatosplenomegaly. Transported for extubation at home. Death within 2 days. **Patient 3:** female, premature at 31 weeks. Consanguineous parents. Birth weight: 1620 g. Dysmorphisms and flexion contractures of the limbs. Respiratory failure on the 5th day of life. On the 14th day of life, the child showed no respiratory movements. Transported for extubation at home. Death after 1 h.
End-of-life care in the pediatric intensive care unit after the forgoing of life-sustaining treatment.	Burns JP et al. (2000)[25]	United States	Prospective, observational study	Describe the attitudes and practices of clinicians (physicians and nurses) in the use of sedation and analgesia in patients at the end of life during the withdrawal of life support measures in the ICU.	Fifty-three patients underwent palliative extubation in intensive care units at three different teaching hospitals in Boston over 18 consecutive months in the mid-1990s.	The median age was 30 months, the median length of stay in the ICU was 13 days, and the median length of stay in the hospital was 18 days. Forty-seven patients (89%) underwent palliative extubation without weaning, and 6 (11%) underwent progressive reduction of MV parameters. In those who underwent weaning from MV, cardiovascular support was also discontinued. Forty-five patients (85%) died within the first 4 hours and 8 (15%) died between 4 and 24 hours. Forty-four patients received sedation and analgesia before support was withdrawn, 47 after support was withdrawn, and 6 patients in a coma did not receive sedatives or analgesics. Only 50% of patients with severe neurological injury received analgesia and sedation compared to 89 to 100% of patients with other clinical conditions. The main reasons for using medications were pain control, anxiety, and dyspnea. All patients used endotracheal tubes.
Home Pediatric Compassionate Extubation: Bridging Intensive and Palliative Care	Zwerdling T et al. (2006)[30]	United States	Case report	Present the case report of a patient who underwent palliative extubation at home based on the development of a care plan.	A 2-month-old male patient with type 1 spinal muscular atrophy was admitted to the ICU with respiratory failure.	A 2-month-old male patient with type 1 spinal muscular atrophy and dependent on mechanical ventilation. He was transported home by the palliative care team (hospice nurse and pediatrician), where he received intravenous benzodiazepine and was extubated. Death occurred 20 minutes after extubation.
Withdrawal of Mechanical Ventilation in Pediatric and Neonatal Intensive Care Units	Munson D (2007)[32]	United States	Case Report and literature review	To present a practical approach to the withdrawal of life support measures and, more specifically, palliative extubation in pediatric and neonatal ICUs.	An extremely premature patient (24 weeks) developed necrotizing enterocolitis and multiple complications due to prematurity, was dependent on MV, and underwent palliative extubation outside the ICU.	Male, 6 months old. Severe sequelae from complications of prematurity, dependent on mechanical ventilation at high settings. Received sedation and analgesia with opioids and benzodiazepines, and reduction of ventilatory settings was initiated. The child presented agitation and dyspnea, and a dose of pentobarbital was administered. Death occurred 40 minutes after removal of ventilatory support. Palliative extubation was performed in the family rest room
Home extubation by a pediatric critical care team: Providing a compassionate death outside the pediatric intensive care unit.	Needle JS (2010)[33]	United States	Case Report and literature review	Present the case report of a patient who underwent palliative home extubation by the pediatric intensive care team.	A patient with Down syndrome and complex congenital heart disease was transported from the ICU for palliative extubation at home.	Male, 6 months old. Patient with Down syndrome, complex congenital heart disease, and sequelae of ischemic stroke. At 4 months, he underwent cavopulmonary anastomosis, but progressed to extubation failure, with no new surgical approach proposed. Transported home for extubation. Received Lorazepam and was extubated in his mother's arms. Death within 2 hours.
Retirada de asistencia respiratoria en domicilio: toma de decisiones en cuidados paliativos pediátricos	Garcia-Salido A et al. (2013)[31]	Spain	Case report	To present a case report of a patient who underwent palliative extubation at home.	A patient with CIUR, prematurity (36 weeks), very low birth weight (1490 g), multiple CNS malformations, atrial septal defect, and severe gastroesophageal reflux disease underwent palliative extubation at home.	Female, 1 year old. Sequelae of prematurity, multiple CNS malformations, atrial septal defect, and severe gastroesophageal reflux disease. Tracheostomized and gastrostomized, dependent on mechanical ventilation. Received analgesic and sedative before palliative extubation. After the procedure, she was given opioids. Death occurred approximately 60 minutes after removal of ventilatory support.
Withdrawal of ventilatory support outside the intensive care unit: guidance for practice	Laddie J et al. (2014)[24]	United Kingdom	Cross-sectional study Retrospective chart review	Develop local guidelines for the practice of palliative extubation outside the ICU based on the analysis of a series of cases.	Fifteen pediatric patients with oncological, neurological, renal, or respiratory diseases who were transferred from the pediatric or neonatal ICU between 2003 and 2012 to settings outside the hospital under the care of the palliative care team in order to undergo palliative extubation.	Fifteen patients with oncological, neurological, renal, or respiratory diseases aged between 2 weeks and 16 years, nine of whom were male, were transferred to their homes (5), hospices (8), or other locations (2) with the respective care plans. The time between admission to the ICU and transfer ranged from 3 to 50 days (mean 14.1 days). The time between withdrawal of ventilatory support and death ranged from “immediately” to 5 days; 12 patients died within 13 hours and only 4 survived more than 2 hours. One of the patients survived after removal of ventilatory support and was discharged from follow-up. Symptoms presented by patients after extubation were: dyspnea (10), agitation (10), pain (9), secretions (9), convulsions (5), and stridor (1), and 3 received some form of oxygen. All patients used endotracheal tubes.
Pediatric Critical Care Transport as a Conduit to Terminal Extubation at Home: A Case Series	Noje C et al. (2017)[26]	United States	Case report	To present the service's experience with the transport of critical patients in palliative care from the ICU for palliative extubation at home.	Three patients aged between 7 months and 18 years with complex chronic conditions were transferred from the ICU to their homes between January 1, 2012, and December 31, 2014, for palliative extubation.	**Patient 1**: male, 18 years old, recurrence of acute lymphocytic leukemia after bone marrow transplant, clinically unstable with multiple organ failure. Extubation and suspension of amines were performed, O_2_ was administered via nasal cannula, and sedation was optimized. Death occurred 15 minutes after extubation. **Patient 2**: male, 6 years old, prostate rhabdomyosarcoma, myeloid leukemia, and severe brain damage as a result of CPR and ECMO use. Extubation was performed and the patient was maintained on room air. Death occurred a few hours after removal of ventilatory support. **Patient 3**: female, 7 months old, type 1 spinal muscular atrophy, hydrocephalus, and neurological sequelae after CPR. Extubation performed at home. Death 15 minutes after extubation. All transports were performed on the same day the decision was made. All patients were on endotracheal tubes and received sedation and analgesia before the procedure.
Interdisciplinary Pediatric Palliative Care Team Involvement in Compassionate Extubation at Home: From Shared Decision-Making to Bereavement	Postier A et al. (2018)[27]	Estados Unidos	Case report	To present the report of two patients who underwent palliative extubation at home and describe the main points to be addressed when considering palliative extubation.	Two patients were transported from the ICU to their homes to undergo palliative extubation.	**Patient 1:** female, 15 years old, with severe anoxic brain injury after a suicide attempt by hanging. Transported to her home to undergo palliative extubation. She was on unspecified medications for comfort. After removal of the endotracheal tube, she showed no signs of discomfort and died after 30 minutes. **Patient 2**: male, 18 months old, with Type I spinal muscular atrophy. Tracheostomized and dependent on mechanical ventilation. After palliative extubation, he survived for 2 or 7 days (data conflicting between text and table) and required opioids to remain comfortable.
Palliative extubation: five-year experience in a pediatric hospital	Affonseca CA et al. (2020)[28]	Brazil	Cross-sectional study Retrospective chart review	To present a series of cases of pediatric patients undergoing palliative extubation in a hospital unit and attempt to identify predictive factors for survival time after the procedure.	Nineteen patients admitted to a pediatric hospital with chronic and irreversible diseases, permanently dependent on ventilatory support, and who underwent palliative extubation between April 2014 and May 2019.	The median age of the patients was 2.2 years, and 58% were female. The median duration of invasive mechanical ventilation was 31 days. Palliative extubation was performed in the ICU in 13 patients and in the inpatient unit in 6. The main symptoms after the procedure were pain and/or dyspnea, which were controlled with the use of opioids and benzodiazepines; 8 patients had no symptoms. Death occurred in 11 patients, with 1 dying during the reduction of ventilatory parameters and the remaining 10 between 15 minutes and 5 days after the procedure. Eight children (42.1%) were discharged from the hospital. No factors were identified that could predict a higher probability of discharge or death after palliative extubation.
Pediatric cardiac critical care transport and palliative care: A case series	Garcia X et al. (2020)[29]	United States	Case report	To present the service's experience with the transport of critical patients in palliative care from the cardiac ICU for palliative extubation at home.	Three patients aged 7 months, 9 months, and 19 years with end-stage heart disease were transported between January 2014 and December 2018 from the cardiac ICU to their homes to undergo palliative extubation.	**Patient 1**: male, 7 months old. Premature, hypoplastic left heart syndrome, underwent multiple surgical procedures with no possibility of cure. Other comorbidities: multicystic renal dysplasia, partial DiGeorge syndrome, and vocal cord paralysis. Transferred for extubation at home under sedation. Received sedation and oxygen via nasal catheter after extubation and died after many years. **Patient 2:** male, 9 months old. Patient with trisomy 21, complete atrioventricular septal defect, underwent surgery at 3 months of age. Multiple postoperative complications. Other comorbidities: hypothyroidism, acute renal failure, adrenal insufficiency, and deep vein thrombosis of the femoral and jugular veins. After transport home, he was baptized in his mother's arms. He was under sedation before and during the extubation procedure. Death occurred after 11 minutes. **Patient 3:** female, 19 years old, with Cockayne syndrome, severe right heart failure secondary to pulmonary hypertension, pleural effusion, and ascites. Transported while on nitric oxide. Required additional sedation due to agonal breathing. Death 2 hours after extubation.
Practice of pediatric palliative extubation in Brazil: a case series	Abath KM et al. (2025)[34]	Brazil	Cross-sectional study Retrospective chart review	To describe the clinical profile, procedures performed, and outcomes of patients undergoing palliative extubation in two intensive care units of a high-complexity teaching hospital.	Twenty-seven patients up to 14 years of age underwent palliative extubation between January 2016 and July 2023.	The median age of the patients was 4 months, and 51.8% were female. Seventy-seven point eight percent of patients had a complex chronic condition. The discussion about palliative extubation had been addressed for 3 patients before admission to the ICU. The main conditions that led to the decision to perform palliative extubation were: severe neurological involvement (70.3%), failure of curative treatment (59.2%), and failure to wean from MV (51.8%). The median duration of invasive mechanical ventilation was 14 days until the indication for palliative extubation and 20 days until the procedure was performed. Sixteen patients received sedatives or analgesics, 13 received dexamethasone, and 4 received medications to control sialorrhea before PE. All patients used endotracheal tubes, and mechanical ventilation was withdrawn without prior weaning in 51.8% of cases. Ventilatory support with O2 or NIV was used in 13 patients after PE. Thirteen patients had symptoms after PE: dyspnea (84.6%), agitation (53.8%), and sialorrhea (11.1%). Twenty-four patients died between 20 minutes and 38 days after PE (median: 3 days).

ICU, intensive care unit; IUGR, intrauterine growth restriction; BW, birth weight; O2, oxygen; MV, mechanical ventilation; NIV, noninvasive ventilation; stroke, cerebrovascular accident; CNS, central nervous system; CPR, cardiopulmonary resuscitation; ECMO, extracorporeal membrane oxygenation; PE, palliative extubation.

Among the 12 selected articles, eight were case reports, [26,27,29, 30, 31, 32, 33,35] and four were cross-sectional studies, [24,25,28,34] three retrospective [24,28,34] and one prospective [25]. Only one study performed a multicenter analysis [25].

The studies were published between 1994 and 2025. Seven were conducted in the United States, [25, 26, 27,29,30,32,33] two in the United Kingdom, [24,35] one in Spain, [31] and two in Brazil [28,34].

Eight studies were considered high quality: five case reports [27,29, 30, 31, 32] and three cross-sectional studies [25,28,34]. The remaining studies were classified as moderate quality: three case reports [26,33,35] and one cross-sectional study [24]. The critical evaluation of the studies is presented in Table 3.

**Table 3:** Assessment of study quality using the JBI Critical Appraisal Tools recommended for case reports and cross-sectional studies.

### Case reports

Among the eight studies, [26,27,29, 30, 31, 32, 33,35] six aimed to perform palliative extubation at home: 4 of them were conducted in the United States, [26,27,29,30] two of them [26,29] with greater emphasis on the transport of critical patients, one in Spain, [31] and another in the United Kingdom [35]. The other two studies presented a case report to illustrate the literature review: one of them, a review on the suspension of life support measures in pediatric and neonatal ICU, [32] and the other, a review on the implementation of palliative extubation at home, [33] both in the United States.

### Cross-sectional studies

There are four cross-sectional studies, [24,25,28,34] one prospective, [25] and three retrospective. [24,28,34] Burns et al. [25] described the attitudes and practices of physicians and nurses regarding the use of sedation and analgesia when withdrawing life support in three centers in Boston (USA) in a sample of 53 patients. Laddie et al., [24] in the United Kingdom, in a single center, evaluated 15 patients to develop local guidelines for the practice of palliative extubation outside the ICU. In Brazil, Affonseca et al. [28] and Abath et al., [34] both in a single center, analyzed, respectively, 19 patients undergoing palliative extubation to describe this population and, if possible, identify predictive factors for survival time after the procedure, and 27 patients undergoing palliative extubation to describe the clinical and epidemiological profile of this population.

### Study population and characteristics

The population of all studies included 129 patients undergoing palliative extubation; however, data were extracted for 128 of them, since in the study by Garcia et al., [29] one of the three patients was older than 18 years and was excluded from the analysis. The age of the patients was described as median in three studies [25,28,34] (n = 99), ranging from 4 to 30 months. In the study by Laddie et al. [24] (n = 15), the age ranged from 2 weeks to 16 years. In the other studies, [26,27,29,30, 31, 32, 33,35] the median age calculated for the 14 patients (11.0%) was 7 months (IQR: 2-12).

Among patients, 58.6% (n = 37) of patients [24,26, 27, 28, 29, 30, 31, 32, 33, 34, 35] were classified as female (49.3%), and 38 patients were male (50.6%); Burns et al. [25] did not report the gender of the patients included in their study (n = 53).

The clinical characteristics of patients undergoing palliative extubation involved severe, irreversible, and/or terminal chronic conditions that varied according to the profile of the service where the studies were conducted. The most frequently identified diagnoses were prematurity with severe complications, complex heart disease, cancer, genetic diseases, complex congenital malformations, neurological diseases associated with hypoxic-ischemic encephalopathy, malformations of the central nervous system, and progressive neurodegenerative diseases.

### Case reports

A total of 15 patients were evaluated: three studies [26,29,35] with three patients each, one study [27] with two patients, and the others [30, 31, 32, 33] with only one patient. In the study by Garcia et al., [29] with three patients, one was 19 years old and was excluded from the sample; thus, the population for analysis of the results consisted of 14 patients.

Of the 14 patients who underwent palliative extubation, 6 (43%) were female and 8 (57%) were male; the age range varied from 14 days [35] to 18 years [26]. It is noteworthy that 11 patients (78.6%) were under two years of age (calculated median of 180 days, IQR: 54.5 to 240.0), 10 of whom were less than one year old [26,27,29, 30, 31, 32, 33,35]. Among these 10 children, 6 were preterm newborns [29,31,32,35] whose gestational age at birth ranged from 24 to 36 weeks. Prematurity was associated with malformations, hypoxic-ischemic syndrome, and complications resulting from gestational age (bronchopulmonary dysplasia, cerebral hemorrhage). The other five children under two years of age were diagnosed with spinal amyotrophy (n = 3) [26,27,30] and 21-trisomy associated with complex heart disease (n = 2). [29,33] In three patients over two years of age, [26,27] the associated clinical conditions were prostate rhabdomyosarcoma, relapsed acute lymphocytic leukemia, and severe cerebral sequelae after attempted suicide.

### Cross-sectional studies

There are four cross-sectional studies, [24,25,28,34] one prospective [25] and three retrospective, [24,28,34] totaling 114 patients.

The median age was reported in 99 patients (86.8%) and ranged from 4 months [34] to 30 months [25,28]. For the remaining 15 patients (13.2%), the age ranged from 2 weeks to 16 years. Information about the sex of the patients was available for only 53.5% of the patients, since in the study by Burns, this information was not available. The objective of the study by Burns et al., [25] in a prospective multicenter study with 53 patients, was to evaluate the medications (class and dose) used during the process of withdrawing life support from patients and to analyze the degree of satisfaction of physicians and nurses with the end-of-life care provided, as well as the degree of agreement between them regarding the justifications for the use of medications after extubation. The main clinical conditions of patients who used analgesics or sedatives were acute respiratory failure (n = 15), postoperative (n = 11), sepsis (n = 8), cancer (n = 8), and neurological disorders (n = 5). In the others retrospective and single-center studies, the main primary diagnoses were: neurological, renal, and respiratory diseases, [24] neurological or neuromuscular diseases, [28] and genetic diseases and malformations (cardiac, CNS, renal, and digestive) [34].

### Location of the procedure and device used

Palliative extubation was performed in a hospital setting in 78.1% of patients (n = 100), [25,28,32,34] mainly in the ICU (n = 93) [25,28,34]. Six patients underwent palliative extubation in the inpatient unit [28] and one patient in a private room [32]. Twenty-eight patients (21.9%) [24,28,32] were transported outside the hospital setting to undergo the procedure: 18 (14.6%) to their homes, [24,26,27,29, 30, 31,33,35] 8 to a hospice, [24] and 2 to “other locations” [24]. Most patients undergoing palliative extubation (n = 119; 93.0%) were on endotracheal tubes, [24, 25, 26, 27, 28, 29,30,33, 34, 35] and nine patients (7.0%) were tracheostomized [27,28,31,32].

### Case reports

Thirteen patients (92.9%) underwent palliative extubation at home, [26,27,29, 30, 31,33,35] and in only one patient, [32] this procedure occurred in the hospital, in a private room. At the time of palliative extubation, 78.6% (n = 11) of patients used an endotracheal tube [26,27,29,30,33,35] and 21.4% (n = 3) used a tracheostomy cannula [27,31,32] as IMV devices.

### Cross-sectional studies

Palliative extubation was performed in the ICU in 93 patients (81.6%). [24,25,28,34] Other places where the palliative extubation took place were in a hospice (n = 8), [24] in the inpatient unit (n = 6) [28], at home (n = 5), [24] and for 2 patients, the location was not specified [24]. An endotracheal tube was used for IMV in most patients (94.7%; n = 108) [24,25,28,34]. The others received IMV through a tracheostomy cannula (n = 6) [28].

### **Duration of IMV and decision-making time until** palliative extubation

Among the 128 patients analyzed, in 52 patients (40.6%) [27,28,33, 34, 35] the median time from IMV to palliative extubation was 20 days (IQR: 14-39; n = 27), [34] 31 days (IQR: 11.5-97; n = 19) [28] or 34.5 days (IQR: 12.5-57.2; n = 6) [27,33,35]. Information was not available for 59.4% (n = 76) of patients [24, 25, 26,29, 30, 31, 32].

The time between decision-making and performing palliative extubation was only observed in 24 patients (18.7%): [26,28,31,33] in 19, the median time was 1 day (IQR: 0 to 4.5), [28] and in 5 patients, there was no precision regarding the time (“a few days” and less than 24 hours) [26,31,33]. For 81.3% (n = 104) of patients, [24,25,27,29,30,32,34,35] this information was not available.

Both times, information (IMV duration and time from decision to procedure) was not available for 56.3% of the patients analyzed (n = 72) [24,25,26,29,30,32].

### Case reports

The information about the duration of IMV until PE was available for six patients (42.9%), [27,33,35] and the median was calculated as 34,5 days (IQR: 10 to 60). For 57.1% (n = 8) of patients [26,29,30,31,32], this time was not reported.

The time elapsed between decision-making and palliative extubation ranged from “less than 24 hours to a few days” for five patients [26,31,33]. For most patients (n = 9; 64.3%), this time was not reported [27,29,30,32,35].

Neither IMV duration nor decision-making to palliative extubation time were reported for four patients (28.6%) [29,30,32].

### Cross-sectional studies

The median times of IMV use until palliative extubation were reported in the two Brazilian studies (n = 46; 40.4%): 20 days (IQR: 14-39) [34] and 31 days (IQR:11.5-97) [28]. In the other studies (n = 68; 59.6%) [24,25], this information was not available.

The time between decision-making and palliative extubation was reported in only one study (n = 19; 16.7%): [28] median of 1 day (IQR: 0 to 4.5).

### Medications used and symptom control

The use of medications prior to palliative extubation was reported in 119 patients (93%), [24, 25, 26, 27, 28, 29, 30, 31, 32, 33, 34] with opioids and benzodiazepines being the drug classes used in 97.5% of these patients [25,28,29,30, 31, 32, 33]. In 22 patients (18.5%), some medication was reported to have been used, but it was not specified [24,26,27,29]. Six patients (4.9%) were in coma and did not use medications before palliative extubation. There was no record of medication use prior to extubation in one of the studies, involving 3 patients (2.3%) [35].

After palliative extubation, 116 patients (90.6%) received some medication for symptom control [24,25,27, 28, 29,31,32,34]. In 72 patients (62.1%), who were already using sedatives and analgesics, the medications were maintained, with or without dose increases. In three studies [24,29,34] (n = 44; 37.9%), the class of medications used for symptom control in 44 patients (37.9%) was not specified. In one study, [30] the patient did not use medications after palliative extubation because they were asymptomatic. In three studies (n = 7), [26,33,35] there was no information on medication use after ventilation wean.

The occurrence of signs/symptoms during and/or after withdrawal of ventilatory support was described in seven studies, [24,25,27, 28, 29,32,34] totaling 118 patients. The most reported symptoms were dyspnea, agitation, pain, and sialorrhea. Two patients had no symptoms after palliative extubation [29,30] and there was no information on signs or symptoms after palliative extubation in 4 studies, totaling 8 patients (6,3%) [26,31,33,35].

### Case reports

In 11 patients (78.6%), [26,27,29,30,31,32,33] there were reports of medication use prior to palliative extubation, with sedatives and analgesics being used in 7 of them; however, in 4 patients, the medications used for comfort were not specified [27,31]. In one study [35] (n = 3), there was no information about the use of medications. The use of vasopressors in the 72 hours prior to palliative extubation was reported for one patient (7.1%) among the three in Noje’s study [26]. In 5 patients, no vasopressors were used in the 72 hours prior to palliative extubation, [27,30, 31, 32] and for six patients (42.9%), this information was not available [26,29,33,35].

Symptoms after palliative extubation were reported in 4 patients. One presented agitation and dyspnea [32] and another presented accumulation of secretions in the respiratory tract [27]. For the other two patients, [27,29] the symptoms of discomfort were not specified. Two patients had no symptoms after palliative extubation, [29,30] and in 4 studies [26,31,33,35] (n = 8), this information was not available.

The use of medication after palliative extubation was reported in 6 patients (42.3%), [27,29,31,32] especially barbiturates, opioids, and atropine. In one patient, [30] medication was not necessary. In 7 patients (50%), there was no information about the use of medication for symptoms control after palliative extubation [26,33,35].

### Cross-sectional studies

Medications were used before palliative extubation in 108 patients, [24,25,28,34] and in 6 patients (5.3%), no medication was used to prepare for the procedure [25]. Opioids and benzodiazepines were mainly prescribed, but the use of other medications such as atropine, scopolamine, and corticosteroids was also described. For 15 patients, the class of medications used was not specified [24].

Two studies described the use of vasopressors in “some patients” [25] and in four patients [34] for a population of 53 patients and 27 patients, respectively. However, it is unclear whether their use was within the 72 hours prior to palliative extubation. This information was not available for 34 patients (29.8%) [24,28].

The main signs/symptoms recorded during and/or after withdrawal of ventilatory support in the cross-sectional studies [24,25,28,34] were agitation, dyspnea, pain, and sialorrhea.

For symptom control, the use of benzodiazepines and opioids, [25,28,34] oxygen, [24,28] and non-invasive ventilation support [28,34] was described.

### Patient outcomes: discharge or death

After discontinuation of respiratory support, 116 patients (90.6%) died, [24, 25, 26, 27, 28, 29, 30, 31, 32, 33, 34, 35] and the time between withdrawal of support and death ranged from “immediately” [24] to 38 days. [34] It is important to note that 12 patients (9.4%) [24,28,34] were discharged.

### Case reports

All patients included in the case reports [26,27,29, 30, 31, 32, 33,35] died after palliative extubation. The median time between palliative extubation and death was calculated as 50 minutes (IQR: 17.5 to 120) for 12 patients [26,27,29,30, 31, 32, 33,35]. Three patients were excluded from the sample calculation due to a lack of accurate information (“a few hours,” “many years” 2 or 7 days) [26,27,29].

### Cross-sectional studies

The outcome of patients undergoing palliative extubation was death in 89.5% (n = 102) [24,25,28,34] and discharge in 10.5% (n = 12) [24,28,34]. The time between palliative extubation and patient death varied between studies. In the study by Burns, [25] 45 patients (84.9%) died within the first 4 hours after discontinuation of ventilatory support, and 8 patients (15.1%) died between 4 and 24 hours after the procedure. In the study by Laddie, [24] 14 patients (93.3%) died between “immediately” and 5 days after palliative extubation. In the study by Affonseca, [28] 11 patients (57.9%) died between 15 minutes and 5 days after IMV removal, and in the study by Abath, [34] 24 patients (88.8%) died between 20 minutes and 38 days after the procedure.

To better describe the characteristics of each study, the reported data are presented in a table appended to this article (Supplementary Material - Table 4).

## Discussion

The main objective of this review was to analyze the available evidences regarding the practice of palliative extubation in pediatrics, as well as the profile of this population, given that data in the literature related to the performance of palliative extubation in pediatric patients are scarce, mainly because it is a practice that is not well standardized in children and has a lower prevalence when compared to palliative extubation in adults. The studies provided some answers to the questions outlined in the review regarding the palliative extubation process in the pediatric population.

Palliative extubation in pediatric patients is a procedure with multiple peculiarities and an underexplored area. The number of studies identified for this review was small, and they had different objectives and heterogeneous results. As noted, most of the available studies were case reports, [26,27,29, 30, 31, 32, 33,35] with a small population ranging from one to three patients in each study, and whose objective, for the most, was to describe the performance of palliative extubation at home, a practice that is even less frequent when compared to what occurs in a hospital set.

Therefore, the information obtained and summarized in this review may contribute to scientific communication, contributing to the development of clinical guidelines based on real-life experiences and contributing to the generation of scientific evidence on this topic.

The main aspects for reflection in these studies were related to careful interprofessional planning of end-of-life care, including resource management, [26,27] bioethical and legal aspects, [30,32,33] logistics and appropriate transportation, [26,27] and networking, including hospital care, primary care, and home care [26,27,33]. The studies also highlighted the importance of shared decision-making between health professionals and families about the possibility of PE being carried outside the hospital environment and all the steps necessary for this practice, [27,29] a subject that is still little discussed. It should be noted that the case reports were all from developed countries and demonstrate the need for interdisciplinary interventions, as well as the implementation of home palliative care (PC) as an integrative approach for children and their families [26,27,31,33]. It is worth noting that a systematic review [36] on pediatric PC at home suggests that the provision of this type of care is associated with a higher probability of death occurring at home. However, the evidence is considered low due to the small number of studies and risk of bias.

Cross-sectional studies, [24,25,28,34] which were fewer in number in this review, presented a more robust population, which improves the reliability of the results; however, they are subject to interference from the primary variable of interest in each study. This effect was observed in the only study [25] which was multicenter, prospective, had the largest population, and stood out from the others in which its objective was to evaluate the medications used during the process of withdrawing life support, as well as the degree of satisfaction of the team with the end-of-life care provided and the degree of agreement between physicians and nurses regarding the justifications for the use of medications after extubation. The main contributions of this study were associated with improvements in end-of-life care provided by adequate management of pain and suffering through the administration of analgesics, as well as accountability for the quality of this care. To this end, the authors of this study emphasized the need for open discussion not only with the family, but also with all members of the team assisting the patient; care measures to be instituted when life support is withdrawn; clear documentation of these decisions; and regularly scheduled case reviews for careful evaluation of care. The authors also highlighted the importance of developing consensus guidelines on the administration of sedatives and analgesics for signs and symptoms of patient suffering during the withdrawal of life support treatment. Other studies [24,28,34] added different reflections on palliative extubation practices that included demographic and clinical data, relevant for the development of local guidelines, but which could certainly contribute to expanding knowledge on the subject. It should be noted that the study by Laddie et al. [24] aimed at performing withdrawal of ventilatory support outside the ICU setting, with specific practical, logistical, and legal considerations for such a situation.

The scientific evidence available in this review, although limited by methodological robustness, supports that pediatric palliative extubation is probably a practice with the potential to alleviate suffering and respect individual and family preferences, provided it is conducted by experienced teams and with detailed planning, as also demonstrated by other authors [37,38].

In the pediatric population undergoing palliative extubation, it is noteworthy that most patients had underlying conditions related to the perinatal period, such as congenital malformations and complications associated with prematurity. This data is consistent with that presented in the global palliative care atlas published by the World Hospice and Palliative Care Alliance, [39] in which 33.9% of patients requiring PC are affected by conditions related to the perinatal period, which highlights the importance of offering pediatric and perinatal PC from pregnancy onwards.

### Place of procedure and device used

The present review found that the hospital, especially the ICU, was the predominant setting for palliative extubation, probably due to the availability of adequate structure and the presence of an interprofessional team skilled in caring for patients on artificial life support, including rapid response to unexpected events and adequate symptom management, with the integration of PC throughout the disease [37]. This review also showed that increased access to PC and hospice care is likely to favor the suspension of life support measures outside the hospital setting. The demographics of death at home versus in the hospital have been changing as the pediatric population with complex chronic conditions and technology dependence begins to use home care while also having access to a PC team and advanced care planning.

Ten of the 12 studies selected for review were conducted in the United Kingdom, [24,35] Spain [31] and the United States, [25, 26, 27,29,30,32,33] countries with very high human development indices (HDI), with the first two ranking among the countries with the highest quality of death indices, as reflected by the provision of PC to the population, availability of access to opioids, public information on PC, training of health professionals in PC, and the existence of public policies focused on PC [40,41]. Among these 10 studies, eight [24,26,27,29, 30, 31,33,35] transferred patients from the ICU to receive PC at home, seeking to fulfill the parents’ wishes and emphasizing the importance of allowing parents to choose where their child will live their last moments, considering aspects related to the preservation of privacy and the feeling of security provided by the environment, [26,27,29] which is corroborated by other authors [42,43]. End-of-life care at home can give parents a greater sense of control over the situation and facilitate the grieving process [44]. Parents of children who chose to provide end-of-life care for their children at home, including palliative extubation, reported the experience as positive and very meaningful because it allowed everyone the opportunity to say goodbye and bring back comforting memories and a sense of accomplishment [42]. Home palliative extubation, with its numerous logistical and ethical challenges, including specialized transportation and bereavement support, is feasible and can be carried out safely with rigorous planning, integrated work by the entire care network, trained staff, and integration with home PC or hospice, always respecting the wishes of the patient and/or their family [26,33,36,45].

Two studies [28,34] selected for this review were published in Brazil, which in 2021 ranked 78th among 81 countries evaluated for quality of death [41] and which, only in 2024, was covered by a national PC policy [46]. In these two studies, [28,34] all cases of PE were carried out in a hospital setting.

Despite the potential benefits, pediatric palliative extubation outside the hospital remains a rare practice, facing significant challenges due to the limitations of support in the out-of-hospital setting, including specialized professionals, equipment, and medications, as well as the lack of preparation among families and their support networks. In addition, there is a need for improvements in policy strategies and spaces such as hospices. [47]

The device used for IMV prior to palliative extubation was predominantly the endotracheal tube [24, 25, 26, 27, 28, 29,30,33, 34, 35] compared to tracheostomy [27,28,31,32]. Little is known about the specific differences, in the context of PC, between choosing an endotracheal tube or tracheostomy. In general, recent guidelines on pediatric ventilatory support emphasize that endotracheal tubes are associated with a higher risk of upper airway obstruction after extubation, mainly due to subglottic edema, and require specific assessment and prevention strategies [48]. There is no robust evidence that the choice of device alters the outcome. However, attention must be paid to the risks involved, which may influence technical and clinical aspects of palliative extubation, including the use of specific medications for upper airway obstruction.

### Duration of IMV and decision-making time until palliative extubation

The median time from IMV to palliative extubation ranged from 20 to 34.5 days, [27,28,33, 34, 35] although this information was not available for most patients in this review [24, 25, 26,29, 30, 31, 32]. It is known that for patients with complex chronic diseases or multiple organ failure, the median duration of IMV can often exceed 14 days, a time defined as prolonged ventilation [49] and lasts even more than 22 days [50]. However, there is no standard minimum time for the use of IMV before performing palliative extubation, as the decision-making process is highly individualized and guided by numerous factors such as diagnosis, prognosis, family values, and the performance of the interprofessional team. The decision on the adequacy of support and palliative extubation is a process without a defined timeframe [38,48,50]. Some evidence [38,50] suggests that prolonged IMV may lead to respiratory muscle dysfunction, accumulation of secretions, and an increased risk of respiratory distress after extubation, which requires advanced symptom control strategies to ensure comfort and dignity in the palliative extubation process. The literature [48,50] also highlights that prolonged IMV is one of the main risk factors for extubation failure, even outside the palliative context, which may lead to a higher probability of severe respiratory symptoms or the need for additional interventions.

Similarly to IMV time, information on the time elapsed between the decision and the withdrawal of ventilatory support was not available for most patients, [24,25,27,29,30,32,34,35] which makes the available information of “a few days and less than 24 hours” [26,31,33] and “median of 1 day”, [28] not representative; however, it does raise questions. The decision-making process for withdrawing life support is complex: it involves careful prognostic assessment, structured communication with the family, consensus among the interprofessional team, and ethical and legal principles. It is recommended that this decision be made jointly and following the care objectives defined with the patient and family, which can take days to weeks, depending on clinical evolution, family context, and the team [48]. Thus, the time is different for each patient, varying widely during the process, from the first conversations to the decision-making, reflecting the need to adapt to the individual context and the pace of understanding and acceptance of the family, which may result in prolonged use of IMV. Once the decision to withdraw IMV has been made, it should be implemented as soon as the detailed planning and communication of the withdrawal process have been completed, ensuring adequate symptom management and ongoing emotional support for the family and team. It is essential to ensure that the transition to comfort care occurs in a compassionate setting and aligned with the best interests of the patient [48]. This time should be sufficient for the patient, team, and family to be adequately prepared, without, however, being too long, since the time prior to the implementation of palliative extubation is likely to be related to moments of great anxiety and distress for everyone involved [51].

### Medications used and symptom control

This review [24, 25, 26, 27, 28, 29, 30, 31, 32, 33, 34, 35] observed the use of medications in the palliative extubation process, and among the medications used, as expected, analgesics and sedatives stood out, especially opioids and benzodiazepines. However, the absence of this information for many patients also requires attention.

Medication management during palliative extubation, mainly with the use of analgesics and/or sedatives before, during, or after the procedure, demonstrates a concern with preventing and relieving pain, dyspnea, and agitation, which are the main symptoms related to the withdrawal of ventilatory support. Anticholinergics and corticosteroids may also be associated according to the needs of each patient, as described by other authors, both in pediatric [52] and adult patients [1,15,18,53, 54, 55].

It is important to note that the appropriate use of medications is not associated with a reduction in survival time after extubation, reinforcing that the focus of care should always be to provide relief from suffering and never to anticipate death [37,52].

### Patient outcomes: discharge or death

Death was the most frequent outcome in this review, [24, 25, 26, 27, 28, 29, 30, 31, 32, 33, 34, 35] occurring within a variable time interval ranging from “immediately” to “38 days” after palliative extubation. Survival time after palliative extubation depends on the patient’s clinical and functional profile and can vary widely [37,38,52]. This review highlights that 9.4% of patients were discharged, [24,28,34] similarly to that observed in adult patients [15,16,55,54,55, 56, 57, 58, 59]. No robust data quantifies this phenomenon in pediatrics. In PC settings, a portion of patients may survive longer, especially when there is residual physiological reserve or the indication for extubation is not associated with immediate terminal organ failure [38]. Unpredictability should be considered, and the possibility of survival after palliative extubation should be anticipated and discussed with the interprofessional team and family to ensure alignment of expectations and adequate preparation for hospital discharge.

### Limitations

Limitations were identified in this review because, although a search strategy was systematically applied to multiple databases, the possibility that relevant studies may have been missed cannot be ruled out. In addition, publication bias may have occurred due to the search for abstracts only in english. Furthermore, information about some variables of interest was not available in all selected studies, which limits the volume of information available, impacting possible conclusions on some topics. Finally, the included studies had diverse objectives and variable and heterogeneous results, making it difficult to report and synthesize the results of all selected studies.

## Conclusion

This review revealed that data on palliative extubation in pediatrics are scarce and that there are disparities in experiences related to the process. The review suggests that when deciding to discontinue life-sustaining care, a complex variety of factors must be considered in clinical practice at the various stages involved in palliative extubation, and that an experienced interprofessional team with advanced training in PC should address common challenges related to planning and managing the process. This review also highlights the need for guidelines and public policies that bring improvements to the entire healthcare system for children and adolescents with life-limiting and life-threatening conditions. Knowledge of aspects related to palliative extubation is relevant, especially when considering the current clinical and epidemiological scenario in which the authors observe a progressive increase in the number of patients living with complex chronic diseases, often with major compromise in functionality and with low quality of life. The authors consider the publication of future studies on the subject to be of great importance.

## Funding

This research did not receive any specific grant from funding agencies in the public, commercial, or not-for-profit sectors.

## Data availability statement

The data that support the findings of this study are available from the corresponding author.

## Conflicts of interest

The authors declare no conflicts of interest.

## Appendix Supplementary materials

Image, application 1
