# Supplementary material for: Palliative extubation in pediatrics: a scoping review
Source: J Pediatr (Rio J). 2025 Nov 12;101(6):101468. doi: 10.1016/j.jped.2025.101468 (PMC12663013; doi:10.1016/j.jped.2025.101468)
Supplement: Supplementary file 1 [file mmc1.docx]

JPED-D-25-00328_ Supplementary Material

| **Table 4** Supplementary Material: Variables extracted from the 12 studies included in the scope review (1994–2025). | | | | | | | | | | |  |
| --- | --- | --- | --- | --- | --- | --- | --- | --- | --- | --- | --- |
| **Title, Author (year of publication)** | **Device used for ventilation** | **Time of mechanical ventilation until PE Time between decision and PE Location of PE** | **Time between decision and PE** | **Location of PE** | **Medications used in preparation for the procedure** | **Use of amines in the 72 hours prior to PE** | **Symptoms observed during and/or after removal of ventilatory support** | **Treatment instituted to control symptoms after PE** | **Outcome after PE** |  |  |
| Withdrawal of neonatal intensive care in the home, Hawdon JM et al (1994)^35^ | Endotracheal tube | Patient 1: approximately 20 days Patient 2: 7 weeks Patient 3: approximately 10 days | ND | At home | ND | ND | ND | ND | Patient 1: Death after 8 hours Patient 2: Death after 2 days Patient 3: Death after 1 hour |  |  |
| End-of-life care in the pediatric intensive care unit after the forgoing of life-sustaining treatment, Burns JP et al (2000)^25^ | Endotracheal tube | ND | ND | ICU | Benzodiazepines in 44 patients (midazolam, lorazepam, or diazepam) and barbiturates in 3 patients. Opioids in 44 patients. Six patients did not receive sedation and/or analgesia (previously comatose) | Some patients did, but this was not specified in the article | Anxiety, pain, and dyspnea | Opioids were initiated in 2 patients. The dose of sedation and analgesia was increased to almost twice the average dose used before withdrawal of ventilatory support | 45 patients died within the first 4 hours after withdrawal of mechanical ventilation, and 8 patients died between 4 and 24 hours |  |  |
| Home Pediatric Compassionate Extubation: Bridging Intensive and Palliative Care, Zwerdling T et al (2006)^30^ | Endotracheal tube | ND | ND | At home | Benzodiazepine (Lorazepam) | No | None | None | Death within 20 minutes after after withdrawal of ventilatory support |  |  |
| Withdrawal of Mechanical Ventilation in Pediatric and Neonatal Intensive Care Units, Munson D (2007)^32^ | Tracheostomy tube | ND | ND | Hospital (family rest room) | Morphine, methadone, and midazolam | No | Agitation and dyspnea | Pentobarbital | Death within 40 minutes after withdrawal of ventilatory support |  |  |
| Home extubation by a pediatric critical care team: Providing a compassionate death outside the pediatric intensive care unit, Needle JS (2010)^33^ | Endotracheal tube | 2 months | Few days | At home | Lorazepam | ND | ND | ND | Death within 2 hours after withdrawal of ventilatory support |  |  |
| Retirada de asistencia respiratoria en domicilio: toma de decisiones en cuidados paliativos pediátricos, Garcia-Salido A et al (2013)^31^ | Tracheostomy tube | ND | Less than 24 hours | At home | Morphine and midazolam | No | ND | Morphine | Death within approximately 60 minutes after withdrawal of ventilatory support. |  |  |
| Withdrawal of ventilatory support outside the intensive care unit: guidance for practice, Laddie J et al (2014)^24^ | Endotracheal tube | ND | ND | At home (n = 5), hospice (n = 8) or other locations (n = 2) | Not specified | ND | Dyspnea (n=10), agitation (n=10), pain (n=9), secretions (n=9), convulsions (n=5), and stridor (n=1) | Three children received oxygen at home. Treatment of symptoms not specified. | Death “immediately” to 5 days after PE. One child survived after removal of ventilatory support and was discharged from follow-up by the palliative care team. |  |  |
| Pediatric Critical Care Transport as a Conduit to Terminal Extubation at Home: A Case Series, Noje C et al (2017)^26^ | Endotracheal tube | ND | Less than 24 hours | At home | Sedatives and analgesics | Yes in 1/3 patients | ND | ND | Death between 15 minutes (2/3 patients) and a few hours (1/3 patient) after withdrawal of ventilatory support. |  |  |
| Interdisciplinary Pediatric Palliative Care Team Involvement in Compassionate Extubation at Home: From Shared Decision-Making to Bereavement, Postier A et al (2018)^27^ | Pacient 1: Endotracheal tube  Pacient 2: Tracheostomy tube | Patient 1: 2 days  Patient 2: 8 months | ND | At home | Medications for comfort not specified | No | Patient 1: accumulation of secretions in the airways  Patient 2: Presented symptoms not specified by the authors | Patient 1: Atropine drops Patient 2: Morphine | Patient 1: 30 minutes after withdrawal of ventilatory support. Patient 2: 2 days or 7 days (there are conflicting data between the text and the table) |  |  |
| Palliative extubation: five-year experience in a pediatric hospital, Affonseca CA et al (2020)^28^ | Endotracheal tube (n=13) or tracheostomy tube (n=6) | Median 31 days (IQR: 11.5–97) | Median 1 day (IQR: 0 to 4.5) | ICU (n=13) ou infirmary (n=6) | Corticosteroids, atropine, scopolamine, opioids. | ND | Dyspnea and/or pain | Benzodiazepines and opioids | Death: 11 patients, between 15 minutes and 5 days after withdrawal of ventilatory support.; discharge: 8 patients |  |  |
| Pediatric cardiac critical care transport and palliative care: A case series, Garcia X et al (2020)^29^ | Endotracheal tube | ND | ND | At home | All three patients were receiving unspecified sedation | ND | Patient 1 - Unspecified discomfort  Patient 2 - None  Patient 3 - Agonizing breathing | Patient 1: Unspecified extra sedation Patient 2: Was previously receiving sedatives  Patient 3: Extra sedation, unspecified | Patient 1: Death after many years.  Patient 2: Death within 11 minutes.  Patient 3: Death within 2 hours |  |  |
| Practice of pediatric palliative extubation in Brazil: a case series, Abath KM et al (2025)^34^ | Endotracheal tube | Median 20 days (IQR: 14–39) | ND | ICU | 16 patients: Unspecified sedatives and analgesics. 13 patients: dexamethasone. 4 patients: anticholinergic. | Yes, in 4 patients | Dyspnea (n=11), agitation (n=7), sialorrhea (n=?) | Ventilatory support with O2 or NIV was used in 13 patients after PE | Death: 24 patients, between 20 minutes and 38 days after withdrawal of ventilatory support; discharge: 3 patients |  |  |
| Abbreviations: ND: no data; PE: paliative extubation; ICU: intensive care unit; NIV: No invasive ventilation | | | | | | | | | | | |
